# Development of an evidence evaluation and synthesis system for drug-drug interactions, and its application to a systematic review of HIV and malaria co-infection

**DOI:** 10.1371/journal.pone.0173509

**Published:** 2017-03-23

**Authors:** Kay Seden, Sara Gibbons, Catia Marzolini, Jonathan M. Schapiro, David M. Burger, David J. Back, Saye H. Khoo

**Affiliations:** 1 Department of Molecular and Clinical Pharmacology, Institute of Translational Medicine, University of Liverpool, Liverpool, United Kingdom; 2 Division of Infectious Diseases and Hospital Epidemiology, Departments of Medicine and Clinical Research, University Hospital of Basel, Basel, Switzerland; 3 National Hemophilia Center, Sheba Medical Center, Ramat Gan, Israel; 4 Department of Pharmacy & Radboud Institute of Health Sciences (RIHS), Radboud University Medical Centre, Nijmegen, the Netherlands; Imperial College London, UNITED KINGDOM

## Abstract

**Background:**

In all settings, there are challenges associated with safely treating patients with multimorbidity and polypharmacy. The need to characterise, understand and limit harms resulting from medication use is therefore increasingly important. Drug-drug interactions (DDIs) are prevalent in patients taking antiretrovirals (ARVs) and if unmanaged, may pose considerable risk to treatment outcome. One of the biggest challenges in preventing DDIs is the substantial gap between theory and clinical practice. There are no robust methods published for formally assessing quality of evidence relating to DDIs, despite the diverse sources of information. We defined a transparent, structured process for developing evidence quality summaries in order to guide therapeutic decision making. This was applied to a systematic review of DDI data with considerable public health significance: HIV and malaria.

**Methods and findings:**

This was a systematic review of DDI data between antiretrovirals and drugs used in prophylaxis and treatment of malaria. The data comprised all original research in humans that evaluated pharmacokinetic data and/or related adverse events when antiretroviral agents were combined with antimalarial agents, including healthy volunteers, patients with HIV and/or malaria, observational studies, and case reports. The data synthesis included 36 articles and conference presentations published via PubMed and conference websites/abstract books between 1987-August 2016. There is significant risk of DDIs between HIV protease inhibitors, or NNRTIs and artemesinin-containing antimalarial regimens. For many antiretrovirals, DDI studies with antimalarials were lacking, and the majority were of moderate to very low quality. Quality of evidence and strength of recommendation categories were defined and developed specifically for recommendations concerning DDIs.

**Conclusions:**

There is significant potential for DDIs between antiretrovirals and antimalarials. The application of quality of evidence and strength of recommendation criteria to DDI data is feasible, and allows the assessment of DDIs to be robust, consistent, transparent and evidence-based.

## Introduction

Antiretrovirals (ARVs) are among the most therapeutically risky agents for drug-drug interactions (DDIs), through their effects on liver metabolising enzymes such as the cytochrome P450 isoenzymes (CYP450) and drug transporters. Also, changes in exposure to ARVs caused by a DDI may result in either development of resistance or drug toxicity, both reducing HIV treatment success. Amongst the different classes, a rank order of DDI potential is protease inhibitors (PIs) & cobicistat >> non-nucleoside reverse transcriptase inhibitors (NNRTIs, excluding rilpivirine) > integrase inhibitors = rilpivirine = maraviroc > nucleoside or nucleotide reverse transcriptase inhibitors (NRTIs).[1–5] The co-administration of contraindicated drugs has been found to account for 5.2% of 209 hospital admissions in the USA in patients receiving ARVs.[6] ‘Clinically significant’ DDIs involving ARVs affect 14–41% of patients in Europe,[3–5, 7] and almost 20% of patients in Kenya and Uganda.[1, 8] A substantial proportion of these carry potential to lower ARV exposure, increasing risk of treatment failure and viral resistance.

Information concerning the safety of co-administering drugs is derived from various sources. Pharmacokinetic drug interaction studies characterise in detail how one drug affects exposure of the other, but are not designed to exclude the risk of any harm resulting, or to capture population diversity in DDIs. Observational cohorts or case reports may identify clinical harms from DDIs, but are subject to a range of potential confounders. When clinicians are confronted by a potential DDI, clinical judgement of risk versus benefit should be informed by best available evidence, yet a framework on which to assess this evidence is lacking.

For example, conventional DDI studies characterise detailed pharmacokinetics on relatively small numbers of human subjects, more often than not in healthy volunteers, with special populations (such as children, pregnancy, liver or renal impairment) excluded. Formulations and doses studied may vary from the final authorised product. Study design e.g. single dose versus steady-state, parallel versus crossover group, pharmacokinetic sampling strategy, use of population pharmacokinetic modelling, differs. For pragmatic reasons, most DDI studies are powered to show a difference in key parameters of drug exposure, rather than clinical endpoints. Consequently, whilst lack of any significant pharmacokinetic impact can exclude a DDI with confidence, the clinical relevance of a change in drug exposure must be interpreted against the therapeutic index of the affected drug, the nature of any resulting harm and the ability to monitor or prevent that harm. These key data are not always known. It is therefore unsurprising that regulatory agencies such as the European Medicines Agency and the Food and Drug Administration occasionally take different views when presented with the same evidence: for example, the combination of atazanavir and boceprevir is not recommended in the boceprevir US Prescribing Information, but the UK Summary of Product Characteristics (SPC) for boceprevir and atazanavir state it can be considered on a case by case basis. Some DDI studies cannot be ethically undertaken, due to prior knowledge of *in vitro* pharmacokinetics, or toxicity profiles of the drugs suggesting significant risk of harm.

The Grading of Recommendations Assessment, Development, and Evaluation system (GRADE) is a transparent and structured process for developing and presenting summaries of evidence, including its quality, as a basis for making recommendations in health care[9] and now represents the internationally accepted benchmark for development of clinical guidelines. Here, we developed criteria, based on the principles of GRADE, to guide therapeutic decision making for DDIs in an area of considerable public health significance: the co-existing global epidemics of HIV and malaria. HIV infection has a considerable impact on malaria, affecting parasitaemia,[10] disease severity (especially in areas of unstable transmission) and mortality during pregnancy.[11] Malaria infection is associated with increased viral burden of HIV.[12] In settings where the highest burden of HIV-malaria co-infection exists, lack of pharmacovigilance structures and laboratory monitoring, coupled with the high background of febrile and other illness may mask harm caused by clinically significant DDIs. Protocol-based ARV treatment and inflexibility of dosing when using fixed-dose combinations of ARVs can make many DDIs harder to manage.

There are no robust methods published for formally assessing quality of evidence relating to DDIs, despite the diverse sources of information. We aimed to define a transparent, structured process for developing evidence quality summaries, in order to guide therapeutic decision making. This was applied to a systematic review of DDI data with considerable public health significance: HIV and malaria, where a range of complex interactions exists, which occur via differing mechanisms.

## Methods

We assessed all studies that evaluated pharmacokinetic data or adverse events when antiretroviral agents were combined with antimalarial agents, including healthy volunteers, patients with HIV and/or malaria; observational studies, and case reports. Drugs in development but not yet licensed at the time of writing (August 2016) were excluded.

The outcomes of interest were adverse clinical events related to co-administration of two or more drugs, or change (or demonstrated lack of change) in pharmacokinetic parameters related to exposure of either antiretroviral or anti-malarial drug when both were co-administered. Adverse clinical events in this context were defined as: a response which is noxious and unintended, where there is a suspicion of a causal relationship between co-administration of the drugs of interest, and the occurrence. Side effects, deranged laboratory parameters and lack of treatment efficacy/treatment failure were included. Pharmacokinetic parameters of interest were area under the concentration time curve, or exposure (AUC), maximum drug concentration (Cmax), drug concentrations at the end of the dosing interval (Cmin), elimination half life (t1/2) and clearance (CL, or apparent oral clearance CL/F) Day 7 dosing concentrations were considered where appropriate. Adverse clinical events were regarded as critical outcomes, and the potential clinical significance of all outcomes was interpreted in the light of existing knowledge about known efficacy and toxicity of the drugs in question.

The development of the research question and outcomes were not informed by patient involvement.

### Study inclusion criteria & search strategy

The following search was used on PubMed (1987-August 2016).

DrugName AND CoMed AND english[Language] NOT review[Publication Type]

We searched the following conference reports for (peer-reviewed) abstracts relating to DDIs:

- Conference on Retroviruses and Opportunistic Infections (2004 –March 2016)
- International AIDS Society Conference (2005 –July 2015)
- International AIDS Conference (2004- July 2016)
- Interscience Conference on Antimicrobial Agents and Chemotherapy (2004—Sept 2015)
- International Workshop on Clinical Pharmacology of HIV Therapy/HIV & Hepatitis Therapy (2004—June 2016)
- International Congress on Drug Therapy in HIV Infection (2004—November 2014)
- European AIDS Clinical Society (2005-October 2015)
- British HIV Association Annual Conference (2002-April 2016)

For all co-prescribed drugs, we searched the manufacturer’s SPC (UK) (http://emc.medicines.org.uk/) and Prescribing Information (USA) (from each ARV manufacturer’s website). Websites were accessed to June 2016). We utilised a standard data extraction template to systematically assess and summarise the evidence, and extracted relevant data into evidence summary templates (see S1 Table). To generate recommendations, manufacturer prescribing information was considered alongside published data, particularly where no data were available.

### Study quality assessment

#### Grading quality of evidence

Grading of quality of evidence was achieved using a methodology adapted from the GRADE system of classification[9] (Table 1).The study design considered to yield the highest quality of evidence for assessing DDIs was an appropriately powered randomised, controlled interaction trial, with full pharmacokinetic sampling and clinical or validated surrogate endpoints.[13] However trials of this nature are rarely pragmatic or feasible; costly, difficult to recruit to and may not be ethically justifiable. Our evidence base was therefore gathered from smaller pharmacokinetic-based studies, retrospective cohort surveys or case reports. Evidence based on population pharmacokinetic modelling was graded according to the quality of the primary data upon which that model was based. Specific examples of issues leading to downgrading were: lack of clinical endpoints (as adverse clinical events are considered a critical outcome), trough, or ‘random’ pharmacokinetic sampling or sparse sampling not supported by a validated population pharmacokinetic approach, use of single dose studies (more acceptable for known enzyme inhibitors, less acceptable for known enzyme inducers), healthy volunteer data where existing literature suggests different plasma drug exposure in disease.

**Table 1:** Stages and criteria for quality of evidence assessment.

1. Establish initial level of quality or confidence		2. Consider lowering or raising level of quality or confidence		3. Final level of quality confidence rating
***Initial confidence in an estimate of effect***	***Study design (examples)***	***Reasons for considering lowering or raising confidence***		***Confidence in an estimate of effect across those considerations***
		**Lower if**	**Higher if**	
**High confidence**	*Randomized trials*	**Risk of Bias** **Inconsistency** **Indirectness** - Lack of clinical endpoints - Dose not in current use - healthy volunteer data where known different plasma drug exposure in disease **Imprecision** -inadequate duration of dosing -inadequate power to demonstrate effect* -Random/sparse PK sampling **Publication bias**	**Large effect** - 50% decrease or 2-fold (100%) increase in AUC (Cmax, Cmin if AUC not studied). - Major clinical or laboratory abnormality	**High (1)**
**Moderate confidence**	*Controlled pharmacokinetic studies*			**Moderate (2)**
**Low confidence**	*Observational studies*		**Dose response** **All plausible confounding & bias** would reduce a demonstrated effect **or** would suggest a spurious effect if no effect was observed	**Low (3)**
**Very low confidence**	*Single case reports*, *knowledge of drug disposition which predicts presence or absence of interaction*, *recommendations in manufacturer’s Prescribing Information*			**Very low (4)**

* N = 10 required in order to have 80% power to show a 50% difference, assuming 50% variation in PK. N = 15 required in order to have 80% power to show a 50% difference, assuming 50% variation in PK.

**Key:** PK: pharmacokinetic, AUC: area under the concentration-time curve, Cmax: peak plasma concentration, Cmin: minimum plasma concentration

Perhaps the most controversial issue is developing criteria for downgrading studies due to insufficient power to exclude clinically significant pharmacokinetic changes. The meaningfulness of any change in pharmacokinetics relates to the magnitude of that change in relation to both the therapeutic index of the drug and its pharmacokinetic-pharmacodynamic relationship. Superimposed on this are differences in pharmacokinetics and susceptibility to toxicity between HIV positive patients and patients with other conditions. Regulatory authorities accept data from relatively small studies (typically n = 12–24) for new drug applications, although these numbers frequently yield <80% power to demonstrate non-bioequivalence. Certainly pharmacokinetic changes just outside bioequivalence are unlikely to be clinically meaningful for most drugs, particularly those with a large background variability in pharmacokinetics (CV>25%). We therefore based our criteria on 2012 draft FDA guidance[14] as follows: For drugs with defined ‘no effect’ boundaries, we interpreted the significance of pharmacokinetic changes with reference to these thresholds (Table 1). For the majority of drugs, where ‘no effect’ boundaries have not been characterised, we took an arbitrary but pragmatic view that any sample size should at least be able to rule out a large change such as a halving or doubling in AUC (assuming a typical CV of 50%), with at least 80% power, recognising that this is an area which still requires further debate. Where AUC was not reported, a similar magnitude of change in Cmax, Cmin or Ctrough was used. For reference, FDA recommendations for drug-interaction studies classify strong inhibitors of CYP450 enzymes as those causing a ≥5 fold increase in AUC or a >80% decrease in CL; moderate inhibitors a ≥2 to < 5-fold increase in AUC or 50–80% decrease in CL; and weak inhibitors cause a ≥1.25 but < 2-fold increase in AUC or 20–50% decrease in CL. Likewise, strong inducers are considered those which cause ≥80% decrease in AUC, moderate inducers cause 50–80% decrease in AUC, and weak inducers cause 20–50% decrease in AUC.[14]

Table 1 gives examples of initial quality levels assigned to study designs, with up- or down-grading specifically applied to drug interaction data. Studies available as abstracts only, or evidence from in-house studies of drug interactions which were not published in peer-reviewed journals were graded as ‘very low’, where insufficient details concerning study design were available. Studies demonstrating a large magnitude of effect, such as a major clinical or laboratory abnormality, as judged by grader consensus, or a large change in pharmacokinetic parameters were upgraded providing there was demonstrable lack of bias. Down-grading was applied for inconsistency, reporting bias and limitations in pharmacokinetic sampling. All gradings were carried out independently by two assessors (KS and SG), and then discussed among a team of four (KS, SG, SK, DJB) in order to reach consensus. All gradings and recommendations were verified by a fifth assessor (CM), in order to obtain expert advice which was independent to the development of the process. Further adjustments were by discussion and consensus. Quality of evidence gradings were assigned to each study, which differs from the GRADE approach, which assesses quality of a body of evidence as a whole, for each recommendation. Assessing each study individually for a large number of interaction pairs facilitated development of the initial confidence levels and upgrade/downgrade criteria. Final quality gradings were then allocated to the body of evidence as a whole for each interaction studied. It should be noted that this classification system was not developed with or sanctioned by the GRADE Working Group, and no GRADE-based classification system currently exists for evaluating DDIs.

#### Strength of recommendation

The strength of recommendation was formed around the question: ‘is it safe to co-administer both drugs?’ The existing University of Liverpool HIV drug interaction key was used to give the strength of recommendation (Table 2). A RED recommendation was assigned where combinations were contraindicated or to be avoided, AMBER for caution (i.e. potentially manageable interaction with increased vigilance, or monitoring, or dose modification), YELLOW for possible or theoretical interactions, where clinical significance is unclear or unlikely; and GREEN where co-administration of both drugs was considered to be safe. We recognised that in patients with multiple co-morbidities, potential interactions could not be altogether avoided, so a distinction between AMBER or YELLOW was made on the basis of likely potential adverse impact of this interaction, and the therapeutic index of affected drug(s). We ensured that recommendations were consistent with the current licensing guidance wherever possible. Where differences exist between FDA and EMA opinions around a DDI, our recommendations reflect the more conservative approach.

**Table 2:** Strength of recommendation key for co-administration of antiretrovirals with anti-malarial drugs.

	Recommendation: *Question*: *Is it safe to administer both drugs*?	GRADE Equivalent
◊	**Green:** No clinically significant interaction, or interaction unlikely based on knowledge of drug metabolism	Strong for
**⌂**	**Yellow:** Possible or theoretical interaction, with clinical significance unclear or unlikely	Weak for
□	**Amber:** Potential interaction that may require close monitoring, alteration of drug dosage or timing of administration	Weak against
○	**Red:** Interaction likely, do not use or use with caution	Strong against
Δ	There are no clear data, actual or theoretical, to indicate whether an interaction will occur	

## Results

**Fig 1:**
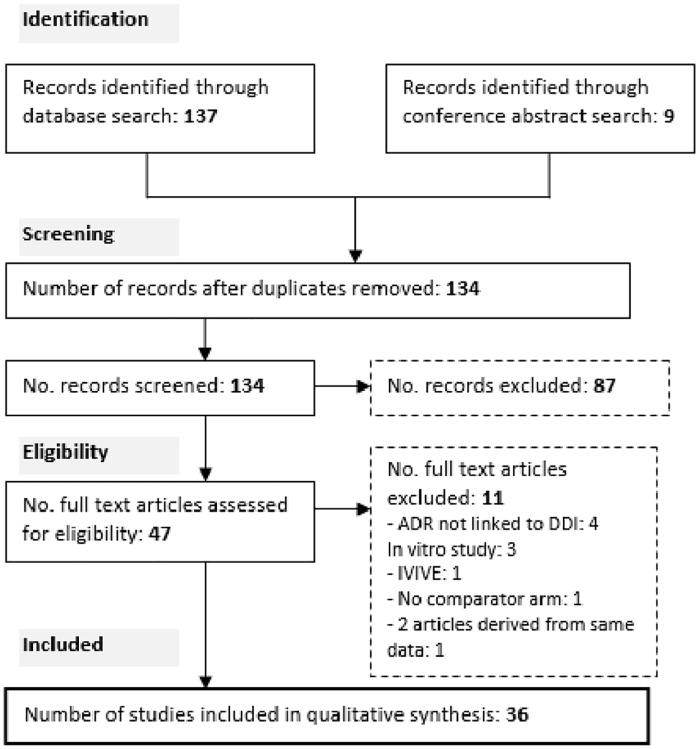
Flow chart illustrating the flow of information through different stages of data collection and synthesis. Key: ADR: adverse drug reaction, DDI: drug-drug interaction, IVIVE: in vitro-in vivo extrapolation

A total of 36 studies and case reports were included (Fig 1). S1 Table summarises the included data which was published, or presented at peer-reviewed scientific meetings. From the 36 studies, 84 quality assessments were made, 43 of which were of low or very low quality. Tables 3, 4 and 5 summarise the recommendation and quality of evidence for each ARV with each anti-malarial drug, incorporating the data summarised in S1 Table and also recommendations made in the manufacturers’ UK SPCs and US Prescribing information.

**Table 3:** Recommendation and quality of evidence table for HIV protease inhibitors/boosting agents and anti-malarial drugs.

	Protease Inhibitors & boosting agents									Cobi (with ATV or DRV)
	ATV/r	DRV/r	FPV/r	IDV/r	LPV/r	NFV	RTV	SQV/r	TPV/r	
Amodiaquine	□ (4)	□ (4)	□ (4)	□ (4)	□ (4)	◊ (4)	□ (4)	□ (4)	□ (4)	◊ (4)
Artemether	□ (4)	□ (2)*	□ (4)	□ (4)	□ (2)*	□ (4)	□ (4)	□ (4)	□ (4)	□ (4)
Artesunate	□ (4)	□ (4)	□ (4)	□ (4)	□ (4)	□ (4)	□ (1)*	□ (4)	□ (4)	◊ (4)
Atovaquone	□ (3)*	□ (4)	□ (4)	□ (4)	□ (2)*	□ (4)	□ (4)*	□ (4)*	□ (4)	◊ (4)
Chloroquine	□ (4)	◊ (4)	◊ (4)	◊ (4)	□ (4)	◊ (4)	◊ (4)	○ (4)	◊ (4)	◊ (4)
Clindamycin	□ (4)	□ (4)	□ (4)	□ (4)	□ (4)	□ (4)	□ (4)	□ (4)	□ (4)	□ (4)
Dihydroartemisinin	□ (4)	□ (4)	□ (4)	□ (4)	□ (4)	□ (4)	□ (4)	□ (4)	□ (4)	□ (4)
Doxycycline	◊ (3)*	◊ (4)	◊ (4)	◊ (4)	◊ (4)*	◊ (4)	◊ (4)	◊ (4)	◊ (4)	◊ (4)
Lumefantrine	□ (4)	□ (1)*	□ (4)	□ (4)	□ (2)*	□ (4)	□ (4)	□ (4)	○ (4)	□ (4)
Mefloquine	□ (4)	□ (4)	□ (4)	□ (4)^$^	□ (2)*	□ (4)*	□ (3)*	□ (4)	□ (4)	□ (4)
Piperaquine	□ (4)	□ (4)	□ (4)	□ (4)	□ (4)	□ (4)	□ (4)	○ (4)	□ (4)	□ (4)
Primaquine	◊ (4)	◊ (4)	◊ (4)	◊ (4)	◊ (4)	◊ (4)	◊ (4)	◊ (4)	◊ (4)	◊ (4)
Proguanil	□ (3)*	□ (4)	□ (4)	□ (4)	□ (3)*	◊ (4)	□ (4)	□ (4)*	□ (4)	◊ (4)
Pyronaridine	○ (2)	○ (2)	○ (2)	○ (2)	○ (2)	○ (2)	○ (1)*	○ (2)	○ (2)	□ (4)
Quinine	□ (4)	□ (4)	□ (4)	□ (4)	□ (2)*	□ (4)	□ (3)*	○ (4)	□ (4)	□ (4)
Sulfadoxine/Pyrimethamine	◊ (4)	◊ (4)	◊ (4)	◊ (4)	◊ (4)	◊ (4)	◊ (4)	◊ (4)	◊ (4)	◊ (4)

**Table 4:** Recommendation and quality of evidence table for HIV non-nucleoside reverse transcriptase inhibitors and newer ARV classes, and anti-malarial drugs.

	NNRTIs				Others			
	EFV	ETV	NVP	RPV	MVC	RAL	DTG	EVG/ Cobi
Amodiaquine	○ (2)*	⌂ (4)	□ (3)	◊ (4)	◊ (4)	◊ (4)	◊ (4)	◊ (4)
Artemether	□ (1)*	□ (2)*	□ (1)*	□ (4)	□ (4)	◊ (4)	◊ (4)	□ (4)
Artesunate	□ (4)	□ (4)	□ (2)*	◊ (4)	◊ (4)	◊ (4)	◊ (4)	◊ (4)
Atovaquone	□ (2)*	□ (4)*	□ (4)	◊ (4)	◊ (4)*	◊ (4)*	◊ (4)	◊ (4)
Chloroquine	□ (4)	◊ (4)	◊ (4)	□ (4)	◊ (4)	◊ (4)	◊ (4)	◊ (4)
Clindamycin	□ (4)	□ (4)	□ (4)	◊ (4)	◊ (4)	◊ (4)	◊ (4)	□ (4)
Dihydroartemisinin	□ (4)	□ (4)	□ (4)	◊ (4)	◊ (4)	◊ (4)	◊ (4)	□ (4)
Doxycycline	□ (3)^$^	□ (4)	□ (3)^$^	◊ (4)	◊ (4)	◊ (4)	◊ (4)	◊ (4)
Lumefantrine	□ (1)*	□ (2)*	□ (2)*	□ (4)	◊ (4)	◊ (4)	◊ (4)	□ (4)
Mefloquine	□ (4)	□ (4)	□ (4)	□ (4)	◊ (4)	◊ (4)	◊ (4)	□ (4)
Piperaquine	□ (4)	□ (4)	□ (4)	□ (4)	□ (4)	◊ (4)	□ (4)	□ (4)
Primaquine	□ (4)	□ (4)	□ (4)	◊ (4)	◊ (4)	◊ (4)	◊ (4)	◊ (4)
Proguanil	□ (4)*	□ (4)*	□ (4)	◊ (4)	◊ (4)*	◊ (4)*	◊ (4)	◊ (4)
Pyronaridine	□ (4)	◊ (4)	□ (4)	◊ (4)	□ (4)	◊ (4)	□ (4)	⌂ (4)
Quinine	□ (4)	□ (4)	□ (3)*	□ (4)	□ (4)	◊ (4)	◊ (4)	□ (4)
Sulfadoxine/Pyrimethamine	◊ (4)	◊ (4)	◊ (4)	◊ (4)	◊ (4)	◊ (4)	◊ (4)	□ (4)

**Table 5:** Recommendation and quality of evidence table for HIV nucleoside reverse transcriptase inhibitors and anti-malarial drugs.

	NRTIs						
	ABC	ddI	FTC	3TC	d4T	TDF	ZDV
Amodiaquine	◊ (4)	◊ (4)	◊ (4)	◊ (4)	◊ (4)	◊ (4)	□ (4)
Artemether	◊ (4)	◊ (4)	◊ (4)	◊ (4)	◊ (4)	◊ (4)	◊ (4)
Artesunate	◊ (4)	◊ (4)	◊ (4)	◊ (4)	◊ (4)	◊ (4)	◊ (4)
Atovaquone	◊ (4)	◊ (4)	◊ (4)	◊ (4)	◊ (4)	◊ (4)	□ (3)*
Chloroquine	◊ (4)	◊ (4)	◊ (4)	◊ (4)	◊ (4)	◊ (4)	◊ (4)
Clindamycin	◊ (4)	◊ (4)	◊ (4)	◊ (4)	◊ (4)	◊ (4)	◊ (4)
Dihydroartemisinin	◊ (4)	◊ (4)	◊ (4)	◊ (4)	◊ (4)	◊ (4)	◊ (4)
Doxycycline	◊ (4)	◊ (4)	◊ (4)	◊ (4)	◊ (4)	◊ (4)	◊ (4)
Lumefantrine	◊ (4)	◊ (4)	◊ (4)	◊ (4)	◊ (4)	◊ (4)	◊ (4)
Mefloquine	◊ (4)	◊ (4)	◊ (4)	◊ (4)	◊ (4)	◊ (4)	◊ (4)
Piperaquine	◊ (4)	◊ (4)	◊ (4)	◊ (4)	◊ (4)	◊ (4)	◊ (4)
Primaquine	◊ (4)	◊ (4)	◊ (4)	◊ (4)	◊ (4)	◊ (4)	□ (4)
Proguanil	◊ (4)	◊ (4)	◊ (4)	◊ (4)	◊ (4)	◊ (4)	◊ (4)
Pyronaridine	◊ (4)	◊ (4)	◊ (4)	◊ (4)	◊ (4)	◊ (4)	□ (4)
Quinine	◊ (4)	◊ (4)	◊ (4)	◊ (4)	◊ (4)	◊ (4)	◊ (4)
Pyrimethamine/sulfadoxine	◊ (4)	◊ (4)	⌂ (4)	⌂ (4)	◊ (4)	◊ (4)	⌂ (4)^$^

Notes: Numbers 1–4 correspond to quality of evidence (Table 1); coloured symbols correspond to the recommendation (Table 2); * denotes drug pairs where data are available (S1 Table); ^$^ denotes drug pairs where data are available, however a recommendation has been made based on manufacturer summaries of product characteristics

The available data for each anti-malarial drug are summarised below.

### Quinine

In one study single dose quinine AUC was increased over fourfold by ritonavir (200mg BD)[15] whereas in others it was halved with lopinavir plus ritonavir (LPV/r 400/100mg BD) with significantly decreased exposure (AUC↓69–99%) to the active metabolite 3-hydroxyquinine. [16, 17] All studies were in healthy volunteers, and since the unbound fraction of quinine is known to be altered with malaria, and both quinine and some HIV protease inhibitors are known to cause ECG changes such as QT prolongation, no change to quinine dosing is recommended, but close monitoring is warranted. Monitoring for ECG changes is also warranted when quinine is administered with rilpivirine and EFV, due to QT prolongation risk.

Quinine exposure (after single dose) is reduced by approximately a third with nevirapine, although the clinical significance is uncertain.[18] A case report in a single patient observed increased *P*. *falciparum* parasitaemia in the presence of quinine in a patient taking nevirapine (attributed to CYP3A4 induction), and switching to atovaquone/proguanil was effective in treating the patient’s malaria.[19] Quinine concentrations were lower, and 3-hydroxyquinine higher, in 6 pregnant women with detectable nevirapine, compared to one patient without. Quinine concentrations were below the therapeutic range in 50% of the patients, however all were successfully treated for malaria.[20] As quinine is an inhibitor of P-gp,[21] an interaction with maraviroc, a P-gp substrate, cannot be excluded. Cobicistat would be expected to increase exposure to quinine via CYP3A4 inhibition.

### Amodiaquine

Significant elevations in hepatic transaminases were reported in the first two healthy volunteers given efavirenz with amodiaquine leading to the premature discontinuation of the study; amodiaquine exposures were significantly increased.[22] A parallel-group comparison in HIV positive patients found that exposure to amodiaquine and its active metabolite were significantly reduced when taken with nevirapine. The mechanism for the interaction is not fully elucidated.[23]

There are no available data for boosted PIs, although caution is advised as protease inhibitors (saquinavir, lopinavir, tipranavir) and some NNRTIs (efavirenz but not nevirapine) are potent CYP2C8 inhibitors[24] *in vitro*, with potential for increased amodiaquine toxicity. In addition, PIs and amodiaquine have each been associated with QT prolongation.[25]

Prolonged neutropenia has been reported in Ugandan children treated with amodiaquine, who were also receiving ARVs, although it is unclear to what extent co-administration with zidovudine may have driven this observation.[26, 27]

### Lumefantrine

Lumefantrine does not seem to prolong the QT interval, but its pharmacokinetics are variable and a marked food effect is observed. The manufacturers advise caution if administering with other drugs which may cause QT interval prolongation, or increase exposure to lumefantrine. Interactions with PIs, NNRTIs and elvitegravir/cobicistat are likely as lumefantrine is a CYP3A substrate, and the manufacturer’s SPC advises that co-administration of CYP3A4 inhibitors such as PIs and cobicistat should be undertaken with caution. An approximately twofold rise in AUC was reported in healthy volunteers who were given lumefantrine with lopinavir/ritonavir,[25] or darunavir/ritonavir[28] and this was confirmed in HIV positive (but malaria negative) patients (lumefantrine AUC increased between 3-fold and 5-fold, 7 day lumefantrine concentration increased up to 10-fold, when compared to ARV naive patients, or those with an EFV-containing regimen).[29, 30] Paediatric patients, but not adults, were found to experience increased adverse events when taking lumefantrine with LPV/r.[31] No change in ECG parameters were observed despite the significantly increased exposure, during single dosing, or a standard six dose regimen.[30, 32] This interaction may be beneficial if it could be shown to reduce the marked pharmacokinetic variability of lumefantrine, or to abolish the food restrictions required. LPV/r increased the exposure to lumefantrine by 439%, but decreased the exposure to dihydroartemisinin by 59.7%. Because of the fixed dose artemether-lumefantrine formulation, no dose adjustment is proposed.[33] In paediatric patients, lumefantrine exposure increased 108%, and 7 day lumefantrine levels were increased 3.4 fold.[34] Lumefantrine exposure increased between 6-fold-10-fold with LPV/r compared to EFV containing ARVs, in HIV positive, malaria positive paediatric patients.[34, 35]

Two well-designed studies demonstrated a decrease in lumefantine exposure (AUC↓56%),[36] and 7day exposure (↓46%)[37] when co-administered with efavirenz at steady state. Etravirine decreased lumefantrine AUC by 13%.[28] Unexpectedly, nevirapine was found to significantly increase exposure to lumefantrine in some studies, [31, 35, 38, 39]

And decrease exposure to lumefantrine in others.[33, 40, 41] Two studies reported no change in lumefantrine exposure (although 7 day lumefantrine concentrations increased in paediatrics) in the presence of nevirapine,[36, 37] and one reports a decrease in nevirapine exposure by 46%.[36] Studies varied in design (healthy subjects, HIV positive vs negative, paediatrics, pharmacokinetic sampling and modelling), although no distinct factor explains the variation in results.

Pharmacokinetic studies in HIV-malaria co-infected children found exposure of artemether and lumefantrine to increase significantly in children taking lopinavir/r as a third agent, compared to efavirenz or nevirapine. HIV positive children taking PI-based regimens have been found to be at reduced risk for malaria compared to NNRTI-based ARVs. Higher rates of malaria treatment failure were observed in children taking efavirenz.[31, 42] 85% of paediatric patients who were taking EFV based ARV regimens had day 7 lumefantrine levels below the treatment threshold. The authors conclude there is a need for alternative dosing of artemether-lumefantrine for children taking EFV-based ARVs.[34]

An observational pharmacokinetic study in adults found that cumulative risk of recurrent parasitaemia was 20-fold higher in patients taking EFV with artemether-lumefantrine, than HIV positive patients taking no ARVs.[40] Dosing adjustments of artemether-lumefantrine were assessed via pharmacokinetic modelling of these data, and an extension of the duration of treatment to five days using the standard dose is suggested.[43] High EFV plasma concentration and CYP2B6*6/*6 genotype have been associated with low lumefantrine plasma concentration and poor malaria treatment response in HIV-malaria- coinfected patients receiving concomitant EFV-based ART. In the absence of concomitant EFV, CYP2B6*6 genotype had no significant influence on lumefantrine plasma exposure.[44] Patients with CYP2B6*6 genotype are at increased risk of QT interval prolongation[45], so monitoring is warranted when co-administering EFV with lumefantrine.

### Artemether

Artemether is metabolised via CYP3A4 to dihydroartemesinin (DHA) (although both compounds have anti-malarial activity, DHA has greater potency). Inhibition of 3A4 would reduce DHA, but increase artemether and potentially increase the short half life of artemether.

In 2 parallel studies, lopinavir/ritonavir reduced exposure to DHA, with artemether increasing in one study, and remaining unchanged in another. A predicted interaction may have been increased arthemeter exposure resulting from lopinavir/ritonavir inhibition of CYP3A4.[29, 30] A crossover study in healthy volunteers observed small decreases in artemether exposure which did not reach statistical significance, and moderate decrease in DHA AUC (↓45%).[25] The observed increased clearance and decreased artemether exposure is likely due to induction of CYP enzymes by lopinavir/ritonavir whereas a decrease in DHA could result from the induction of glucuronidation by ritonavir. No significant changes in QT interval were observed in a single dose study of artemether/lumefantrine and steady state lopinavir/ritonavir.[32] Darunavir/ritonavir was found to decrease steady state artemether and DHA AUC by 16% and 18% in a crossover study in healthy subjects.[28]

Nevirapine was found to decrease exposure to artemether, with little effect, or small decrease in DHA exposure.[36, 39, 41] One crossover study observed a significant decrease in NVP exposure (AUC↓46%),[36] possibly due to artemether induction of CYP3A4, or the effects of nevirapine CYP3A4 autoinduction. In paediatric patients, NVP reduced artemether AUC by 64%, and DHA AUC by 30%.[34]

Efavirenz significantly reduced exposure to artemether and DHA (AUC ↓79%, ↓75% respectively) in HIV positive patients.[36] A smaller healthy volunteer study found no significant reduction in artemether AUC, and a moderate decrease in DHA exposure, although large variability in artemether pharmacokinetics were observed.[37]

Population pharmacokinetic modelling using pooled data[29, 36] proposes the following dose modifications for artemether-lumefantrine: efavirenz decreased exposure to lumefantrine and dihydroartemisinin by 69.9% and 71.7%, respectively. A 250% increase in dose would achieve exposures similar to those without concomitant HIV treatment. Etravirine co-administration reduced artemether AUC by 38% and DHA AUC by 15% at steady state.[28] NVP decreased the exposures to lumefantrine and dihydroartemisinin (25.2% and 41.3%, respectively). A 75% artemether-lumefantrine dose increase would result in adequate exposures. Cobicistat could potentially increase plasma levels of artemether via enzyme inhibition.

### Artesunate

Artesunate is metabolised via hydrolysis and CYP2A6, whereas DHA is predominantly glucuronidated via UGT1A9 and 2B7. Nevirapine has been found to increase overall exposure to artesunate, with DHA pharmacokinetics remaining largely unchanged, with the exception of a decreased DHA:artesunate AUC ratio.[46] No treatment-limiting adverse events were observed in this trial of HIV positive patients stable on nevirapine-based ARVs. However, DHA total exposure was shown to be approximately 2-fold higher in patients with active malaria than healthy volunteers.[47]

Ritonavir moderately increased artesunate AUC (27%), with little effect on Cmax. The effect is thought to be minimal, particularly in the context of variable inter-subject artesunate pharmacokinetics. Decreased exposure to DHA in the presence of ritonavir was observed (AUC↓38%, Cmax↓27%), which may be explained by induction of UGT enzymes by ritonavir.[42] Lopinavir/ritonavir increased artesunate AUC by 80%, and decreased DHA AUC24h by 58.2%.[48]

Co-administration of pyronaridine/artesunate substantially increased exposure to ritonavir. *In vitro* data demonstrate that pyronaridine may inhibit CYP2D6 metabolism and P-gp efflux transport of ritonavir. Coadministration of ritonavir and pyronaridine/artesunate appear to have increased the likelihood of liver enzyme elevations which could not be accounted for by the observed pharmacokinetic parameters associated with co-administration.[42]

### Dihydroartemisinin

Ritonavir and other PIs including atazanavir are unlikely to affect conversion of DHA to the inactive α-DHA-β-glucuronide, as *in vitro* studies have demonstrated that they do not significantly inhibit UGT1A9 or 2B7.[49] Induction of glucuronidation by ritonavir or cobicistat however, cannot be ruled out.

### Mefloquine

Mefloquine had variable effect on ritonavir metabolism: no interaction was noted after a single dose but ritonavir AUC was reduced by 31% and Cmax by 36% after multiple dosing. A decrease in bioavailability appears responsible for the reduced levels of ritonavir.[50]

Pharmacokinetics of mefloquine were not significantly influenced by ritonavir.[50] However, months of concurrent therapy would be necessary for the full extent of an interaction to be reported due to the long half-life of mefloquine. Lopinavir/ritonavir decreased mefloquine AUC 48h by 28.7%. Lopinavir exposure was unchanged, while Cmax was decreased by 22%. Ritonavir AUC24h decreased 44.6%, when administered with artesunate-mefloquine.[48]

A case study including pharmacokinetics in a single patient reported an absence of toxic or sub-therapeutic levels when mefloquine was taken with a regimen containing indinavir and nelfinavir.[51] Cobicistat may potentially increase exposure of mefloquine via CYP3A4 inhibition, whereas EFV, NVP and ETV, may decrease mefloquine exposure via induction of CYP3A4. Mefloquine should be administered with caution with other agents which prolong the QT interval.

### Proguanil

Proguanil is a pro-drug and is partially activated (CYP2C19) to cycloguanil. Interactions may be complex, since synergy with atovaquone is related to proguanil, not cycloguanil.

A parallel group study found that in HIV positive patients taking ATV/r-containing regimens, proguanil AUC decreased by 41% compared to healthy volunteers. Similarly with efavirenz-containing regimens, proguanil AUC decreased 43%, and a decrease of 38% was observed with lopinavir/r.[52] A crossover study in healthy volunteers demonstrated a significant increase in proguanil exposure (AUC↑113%), and a decrease in cycloguanil exposure (↓38%) when administered with efavirenz at steady state.[53] A study in healthy subjects found that lopinavir/r potently induced CYP2C19 activity.[54] The clinical relevance of altering exposure of proguanil in relation to cycloguanil is not known.

### Atovaquone

Atovaquone decreases zidovudine oral clearance leading to a 35% increase in plasma zidovudine AUC, possibly due to inhibition of zidovudine-glucuronide formation. The clinical significance is unknown, and no dose modification is recommended.[55, 56]

Lopinavir/r, atazanavir/r and efavirenz have been found to decrease plasma concentrations of atovaquone. The clinical significance is unknown, however, increases in atovaquone doses may be needed.[52] In a crossover study of HIV positive subjects, Subjects on efavirenz-based ARVs had 47% and 44% lower atovaquone exposure than subjects with no ARVs, at atovaquone doses of 750mg twice daily and 1500mg twice daily, respectively. These doses of atovaquone related to treatment of pneumocystis pneumonia and toxoplasmosis, and differed from regimens used in the treatment or prophylaxis of malaria. In this study, atazanavir/r did not significantly affect exposure to atovaquone.[57]

Atovaquone lowers indinavir exposure, reducing Cmin by~23%. Another healthy volunteer study observed indinavir AUC decrease of 5%, but increase in atovaquone AUC (13%) and Cmax (16%) when both drugs were co-administered. No dosage adjustments are necessary for atovaquone when given with indinavir. The clinical significance of lowered indinavir concentrations is uncertain since these were healthy volunteer studies carried out without ritonavir boosting. Moreover, clinical studies have shown higher plasma indinavir in Thai patients (who have lower body weight), and given the toxicity of indinavir at higher doses, dosage adjustments are not indicated for indinavir/r when dosed with atovaquone or malarone.[30]

A case study in a single patient demonstrated a significant increase in etravirine and unboosted saquinavir exposure (AUC↑55% and 274% respectively) when prophylaxis with atovaquone and proguanil was initiated in a stable patient. No adverse events were reported. Slight, and clinically significant decrease in maraviroc and raltegravir exposure were observed, however no dose alteration is required.[58]

### Piperaquine

Piperaquine is an inhibitor of CYP3A4. It is metabolised by CYP3A4 *in vitro*, however the contribution of CYP3A4 to *in vivo* elimination is unknown. Piperaquine undergoes a low level of metabolism by CYP2C19, and is also an inhibitor of this enzyme. Piperaquine may therefore increase exposure to NNRTIs, PIs, rilpivirine and maraviroc. Exposure to piperaquine may be increased by PIs and cobicistat via CYP3A4 inhibition; or decreased by nevirapine or efavirenz via CYP3A4 induction. A PK study nested in a clinical trial of malaria prevention in pregnant women found that piperaquine exposure and day 7–21 levels were significantly decreased in patients taking EFV-containing ARVs. The efficacy data is yet to be reported.[59] Piperaquine may lead to prolongation of the QT interval and should be used with caution in patients taking agents which also have potential to prolong QT interval, such as PIs EFV and rilpivirine.[37] A study in a cohort of Ugandan paediatric patients has concluded that treatment with dihydroartemisinin and piperaquine alongside LPV/r or NNRTIs is safe and effective.[60] Piperaquine may potentially increase exposure to dolutegravir and elvitegravir via CYP3A4 inhibition, although the clinical significance is unknown.

### Pyronaridine

A parallel group study of healthy volunteers found that artesunate/pyronaridine increased exposure to ritonavir 3-fold, with no change in pyronaridine exposure. This was associated with discontinuations due to increased liver enzymes.[42] Cobicistat as a boosting agent, may be less susceptible to significant interaction with pyronaridine, as CYP2D6 is a minor route of cobicistat metabolism, however an interaction cannot be ruled out.[8] As liver toxicity including significant rises in ALT and AST have been observed with pyronaridine treatment, monitoring of liver function may be advised if using alongside nevirapine or efavirenz. Falls in haemoglobin have also been observed, which may be exacerbated by concurrent treatment with zidovudine[35] As pyronaridine is an inhibitor of P-gp,[61] interaction with maraviroc, or dolutegravir, which are P-gp substrates, cannot be excluded.

### Doxycycline

In an observational pharmacokinetic study, doxycycline did not affect trough concentrations of atazanavir (boosted or unboosted) efavirenz, lopinavir/r or nevirapine.[62] Although no data are available with NNRTIs and doxycycline pharmacokinetics, manufacturers of doxycycline caution that hepatic enzyme inducers may accelerate the decomposition of doxycycline, decreasing its half-life.[39]

### Chloroquine

Although no pharmacokinetic studies concerning concomitant administration with ARVs are available, clinically significant pharmacokinetic drug interactions are unlikely with chloroquine, due to multiple mechanisms of metabolism and elimination. However, caution is warranted if chloroquine is administered with other agents that may prolong the QT interval, such as ATV/r, LPV/r, rilpivirine and efavirenz. [47]

### Clindamycin

*In vitro* studies have demonstrated that clindamycin is predominantly metabolised via CYP3A4. In this analysis, ketoconazole, a CYP3A4 inhibitor, markedly inhibited clindamycin S-oxidase formation.[63] However, there are no available clinical data investigating the effect of ARVs, or other CYP3A4 inhibitors or substrates on clindamycin exposure, therefore the clinical relevance is unknown. Clindamycin inhibited CYP3A4 by approximately 26% *in vitro*.

### Primaquine

The metabolism and elimination of primaquine has not been fully elucidated, and *in vitro* studies have differed in their findings. It has been reported that metabolites formed by CYP2E1, CYP2B6, CYP1A2, CYP2D6 and CYP3A4 variably contributed to the haemotoxicity of primaquine. Significant inhibition of primaquine haemotoxicity by inhibitors of CYP2B6, CYP2D6 and CYP3A4 was observed.[64] Another study reported that CYP2D6 and monoamine oxidase-A are the key enzymes associated with primaquine metabolism, with CYP2D6 mediated pathways playing a main role in efficacy and haemolytic toxicity.[65] The effect of ARVs which may inhibit or induce these enzymes is unknown, however efficacy and toxicity of primaquine should be monitored when co-administed with PIs or NNRTIs.

### Pyrimethamine/Sulfadoxine

Pyrimethamine may depress folate metabolism in patients taking agents associated with myelosuppression, such as zidovudine.[46] Pyrimethamine did not affect zidovudine exposure at doses used to treat cerebral toxoplasmosis.[66] Potentially, pyrimethamine may reduce the renal clearance of emtricitabine and lamivudine, as *in vitro* data suggest that pyrimethamine inhibits renal transport via the multidrug and toxin extrusion transporter MATE1.[67, 68]

## Discussion

We sought to define for the first time a benchmark for studying DDIs, and applied evaluations to study quality in order to achieve transparent and consistent recommendations for managing HIV-antimalarial drug interactions. We have shown that application of a quality of evidence guideline recommendation approach to managing DDIs is feasible. Our benchmark was rarely met, with only 6 of 400 interaction pairs studied being of ‘High’, and 20 of ‘Moderate’ quality. This highlights the neglected nature of this area, and the need for well-designed pharmacokinetic studies, which include clinical endpoints. Design and execution of studies is only one of a number of considerations. Clinical judgements about co-administration of medicines weigh potential for benefit against risk of harms, and DDI studies typically utilise pharmacokinetic exposure as a proxy for this risk, despite lack of requisite knowledge about the concentration-toxicity relationship of the affected drug in many cases. An ‘Amber’ or ‘Yellow’ grading on our traffic light system acknowledges this balance, and allows clinicians to make decisions which are appropriate for their individual setting, taking into account ability to dose modify, to monitor or to prescribe available alternative medications.

There is significant risk of DDIs between HIV PIs, NNRTIs (excluding rilpivirine) and artemesinin-containing antimalarial regimens. The mechanisms for drug interactions were pharmacokinetic (through enzyme inhibition or induction), or pharmacodynamic (eg overlapping toxicities such as QT prolongation, anaemia and hepatotoxicity), or both.

Existing DDI databases often yield incomplete (based solely on manufacturer’s data) or else discordant recommendations, but there are also limitations to using our approach. DDI study designs vary considerably even when designed to support filing of new drug applications. Doses and formulations used may not reflect the final marketed product. Sample size calculations may be unclear. Use of healthy volunteers is the norm, yet the pharmacokinetics of some antimalarials alter with disease. For example, the protein binding and plasma half-life of quinine increases with severity of malaria,[69] lumefantrine absorption is decreased during acute malaria[70] and the pharmacokinetics of mefloquine alters with disease.[71] Concentrations of quinine and lumefantrine also accumulate with multiple dosing, and single dose studies only yield limited data. In addition, pharmacogenetic effects are seldom explored in small drug interaction studies, for example proguanil is predominantly metabolised by CYP2C19, and the frequency of poor metabolisers differs between Africans (3%), South East Asians (20%) and Caucasians. Studies in CYP2C19 poor metabolisers and extensive metabolisers have shown that genetic variants in CYP2C19 can impact the parent drug levels of parent drug and/or metabolite.[72]

Applying quality of evidence ratings to recommendations in this manner allows consistent and transparent recommendations to be made, in a similar manner to guidelines for healthcare interventions. This method applies a quality of evidence rating, which is independent from the clinical recommendation. For example, a low quality evidence source (manufacturer recommendation without data) may lead to a strong recommendation against administration of two drugs, if that recommendation is that co-administering the pair of drugs, even in a clinical study, poses a serious health risk. However, in some cases, evidence of low quality may naturally predispose to a conservative clinical recommendation, where available evidence is not sufficient to confidently rule out an interaction (for example, a study rated as ‘very low’ which concludes there is no interaction, may prompt an ‘Amber’ or ‘Yellow’ recommendation, if there are flaws in the study design that raise sufficient doubts about the results).This is the only systematic review of the data concerning DDIs between ARVs and antimalarials in current usage. There were previously no robust methods for formally assessing quality of evidence relating to DDIs, despite the diverse sources of information. This is the first report of the definition and development of quality of evidence and strength of recommendation categories, and their application to a systematic review of DDI data.

Perhaps the most significant interaction is between amodiaquine and efavirenz, where a healthy volunteer study was prematurely discontinued due to drug-induced liver injury. Consequently, the combination of efavirenz with amodiaquine is contraindicated. This poses a significant problem for many countries in sub-Saharan Africa where amodiaquine-artesunate is a first line antimalarial, since WHO guidelines recommend efavirenz-containing regimens as first line treatment. Whilst many antimalarials undergo P450 metabolism, interactions between boosted protease inhibitors and amodiaquine-, piperaquine- and lumefantrine-containing regimens are manageable through increased pharmacovigilance, and current evidence does not support withholding these treatments given the relatively brief duration of therapy, and adverse consequences of malaria.

A potential concern is the marked reduction in artemether (and lumefantrine) concentrations with efavirenz. Artemesinins are common to all first line combinations, and are metabolised by CYP3A4 and 2B6, and efavirenz predominantly by CYP2B6. It is unclear whether or how polymorphisms in CYP2B6 (which are relatively frequent in many African populations) impact on this interaction. While population pharmacokinetic modelling[33] and *in vitro-in vivo* extrapolation[73] can predict rational dose adjustments, clinical studies are required to determine efficacy and acceptability.

Drug-drug interactions are one of the commonest causes of medication error in developed countries, and are likely to limit efficiency of treatment programs in lower resource settings. Developed country settings may utilise therapeutic drug monitoring, clinical or laboratory monitoring for toxicities, or aim to switch ARVs or co-medications in order to optimise treatment for HIV and other conditions. However, recognition of important DDIs and accurate medication history taking is vital in order to appropriately recognise and manage DDIs. Recognition and management may be aided by electronic clinical support resources, and increasingly via alerts associated with electronic prescribing. Practical steps that can be instituted to reduce the risk of adverse outcomes from DDIs in lower resource settings include integrating national treatment programmes for HIV and other diseases (with protocols that minimise drug interactions), establishing regional networks for pharmacovigilance, and improving the quality of prescribing through training and education of health care workers. Knowledge of common interactions involving antiretrovirals on a country-specific basis will allow targeted training, monitoring and protocol development.

Many recommendations were made based on manufacturers' prescribing information, which in part, explains the high proportion of ‘low’ and ‘very low’ quality gradings, as often the manufacturers’ recommendations are made without presentation of data, or details of how studies were carried out. It is therefore important that DDI and pharmacokinetic studies undertaken by manufacturers are published, or the details of study design are given in product information. This method for summarising data, assigning quality of evidence and strength of recommendation for DDIs was developed, using principles from GRADE, to allow transparent, consistent and evidence-based recommendations for managing DDIs. This systematic review demonstrates the diverse sources of information and range of study design which are used to determine whether co-administration of drugs is safe. For the majority of drug pairs in this review, either no evidence, little evidence, or low quality evidence was available. Further consensus on the gold standard and minimum requirements of drug interaction studies is required.

## Supporting information

S1 Fig PRISMA statement. (TIFF)

S1 Table Summary evidence template for drug-drug interactions between antiretrovirals and antimalarials. (DOCX)
