# Supplementary figures and images for: Development of an evidence evaluation and synthesis system for drug-drug interactions, and its application to a systematic review of HIV and malaria co-infection
Source: PLoS One. 2017 Mar 23;12(3):e0173509. doi: 10.1371/journal.pone.0173509 (PMC5363796; doi:10.1371/journal.pone.0173509)

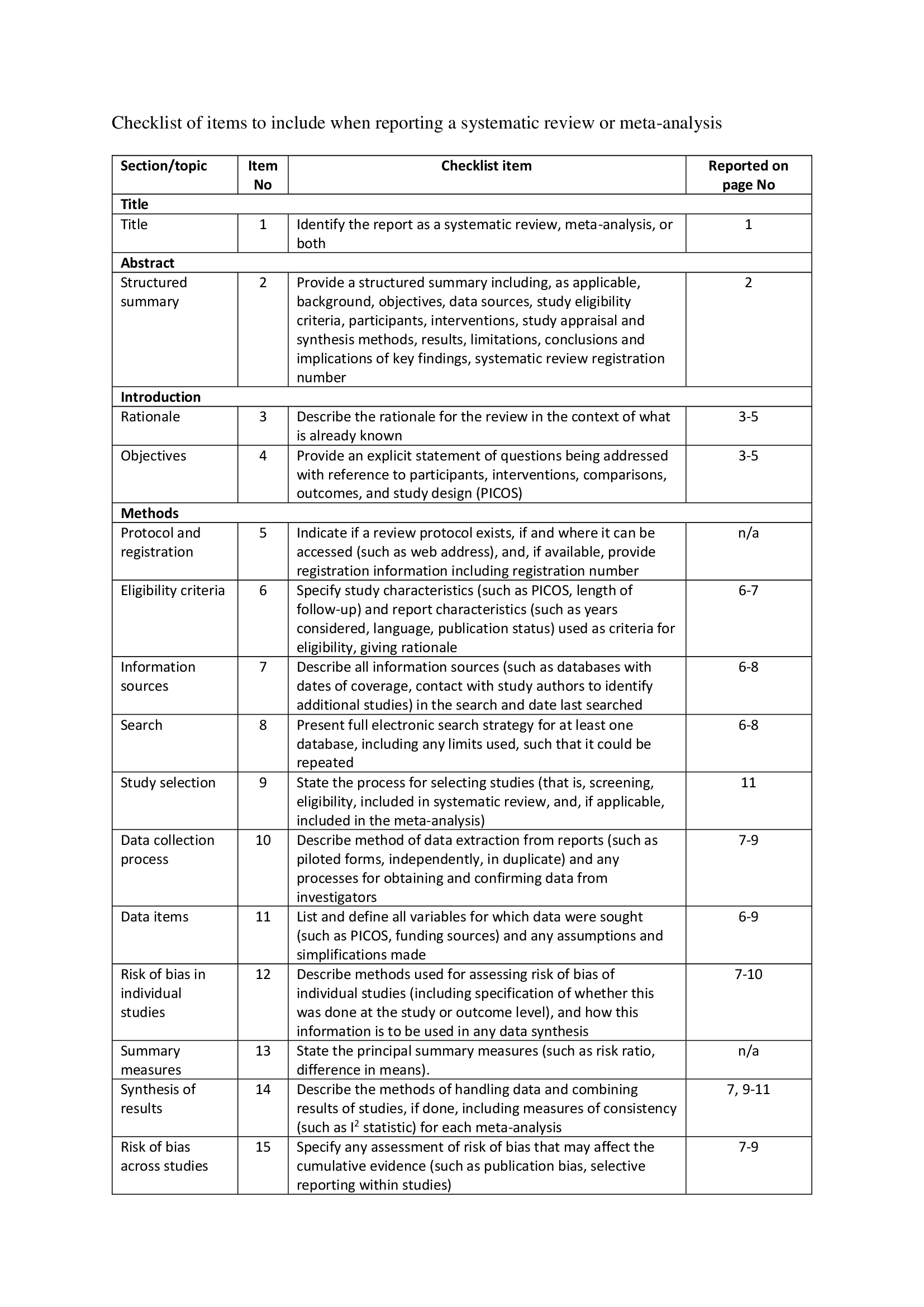

Supplement: S1 Fig — (TIFF) [file pone.0173509.s001.tiff]
