# Supplementary material for: Development of an evidence evaluation and synthesis system for drug-drug interactions, and its application to a systematic review of HIV and malaria co-infection
Source: PLoS One. 2017 Mar 23;12(3):e0173509. doi: 10.1371/journal.pone.0173509 (PMC5363796; doi:10.1371/journal.pone.0173509)
Supplement: S1 Table — (DOCX) [file pone.0173509.s002.docx]

**S1 Table.** Summary evidence template for drug-drug interactions between antiretrovirals and anti-malarial drugs

| **Author, Year** | **ARV** | **Co-Med** | **Study Design & Dosing** | **Subjects** | **Outcomes** | **Results** | **Notes** | **Grade/ Quality** |
| --- | --- | --- | --- | --- | --- | --- | --- | --- |
| Van Luin et al. 2010 | ATV/r | Atovaquone | Open label, multicentre PK study in parallel groups (HIV+ stable on ARVs containing ATV/r 300/100mg OD vs healthy volunteers)  Atovaquone/proguanil 250/100mg single dose | HIV+ on ATV/r n=19  Healthy n=18 | Atovaquone PK | AUC↓46%, Cmax↓49% |  | Low |
|  |  | Proguanil | Open label, multicentre PK study in parallel groups (HIV+ stable on ARVs containing ATV/r 300/100mg OD vs healthy volunteers)  Atovaquone/proguanil 250/100mg single dose | HIV+ on ATV/r n=19  Healthy n=18 | Proguanil PK | AUC↓41%, Cmax↔ |  | Low |
| Calderon et al. 2016 | ATV/r | Atovaquone | Randomised crossover study with washout. Atovaquone 750 mg BD, 14 days, followed by atovaquone 1500 mg BD, 14 days in patients stable on ARVs, or no ARVs. 12hr PK on day 14 | HIV+ (n=29)  EFV (n=10) ATV/r (n=10) no ARVs (n=10) | Atovaquone AUC  Cavg | Atovaquone AUC ↔  Atovaquone Cavg↔  (Patients taking ATV/r compared to patients taking no ARVs) | Dose differs from malaria treatment/prophylaxis regimen | Low |
| Abgrall et al 2013 | ATV, ATV/r | Doxycycline | Observational PK study in patients stable on ATV/r containing ARVs, initiating prophylaxis with doxycycline | HIV+  ATV n=1  ATV/r n=14 | ATV Ctrough | ATV Ctrough↔ |  | Low |
| Kakuda et al. 2013 | DRV/r | Artemether | Randomised 2-phase crossover PK study. Treatments (A and B) in random order, with 4 week washout: A: artemether /lumefantrine 80/480 mg BD alone, 3 days; B: darunavir/ritonavir 600/100 mg BD for 21 days  with artemether/lumefantrine 80/480 mg BD from day 8 (3-days). | Healthy n=14 | Artemether PK  DHA PK  DRV/r PK | Artemether AUC↓16%  DHA AUC↓18%  DRV/r PK↔ | No drug-related serious adverse events reported | Moderate |
|  |  | Lumefantrine | As above | As above | Lumefantrine PK  DRV/r PK | Lumefantrine AUC↑2.75 fold  DRV/r PK↔ | As above | High |
| Kakuda et al. 2013 | ETV | Artemether | Randomised 2-phase crossover PK study. Treatments (A and B) in random order, with a 4 week washout . A: artemether /lumefantrine 80/480 mg BD alone, 3 days; B: etravirine 200 mg BD for 21 days with artemether /lumefantrine 80/480 mg BD from day 8 (3 days). | Healthy n=14 | Artemether PK  DHA PK  ETV PK | Artemeter AUC↓38%, Cmax↓28%, Cmin↓18%  DHA AUC↓15%, Cmax↓16%, Cmin↓17%  ETV PK↔ | No drug-related serious adverse events reported | Moderate |
|  |  | Lumefantrine | As above | As above | Lumefantrine PK  ETV PK | Lumefantrine AUC↓13%  ETV PK↔ | As above | Moderate |
| Tommasi et al. 2011 | ETV | Atovaquone | Case report of a patient with multi-drug resistant HIV infection treated with a salvage regimen of MVC 150mg BD, RAL 400mg, ETV 200mg BD and unboosted SQV 1000mg BD who started atovaquone/proguanil 250/100 mg OD prophylaxis | n=1 | ETV PK | ETV AUC↑55% | No adverse events or laboratory abnormalities | Very Low |
|  |  | Proguanil | As above | As above | As above | As above | As above | Very Low |
| Calderon et al. 2016 | EFV | Atovaquone | Randomised crossover study with washout. Atovaquone 750 mg BD, 14 days, followed by atovaquone 1500 mg BD, 14 days in patients stable on ARVs, or no ARVs. 12hr PK on day 14 | HIV+ (n=29)  EFV (n=10) ATV/r (n=10) no ARVs (n=10) | Atovaquone AUC  Cavg (average steady state concentration) | Subjects on EFV-based ARVs had 47% and 44% lower atovaquone exposure than no ARV subjects at atovaquone doses of 750mg BD and 1500mg BD, respectively | Dose differs from malaria treatment/prophylaxis regimen | Low |
| Van Luin et al. 2010 | EFV | Atovaquone | Open label, multicentre PK study in parallel groups (HIV+ stable on ARVs containing EFV 600mg OD vs healthy volunteers)  Atovaquone/proguanil 250/100mg single dose | HIV+ on EFV n=20  Healthy n=18 | Atovaquone PK | Atovaquone AUC↓75%, Cmax↓44% |  | Moderate |
|  |  | Proguanil | Open label, multicentre PK study in parallel groups (HIV+ stable on ART containing EFV 600mg OD vs healthy volunteers)  Atovaquone/proguanil 250/100mg single dose | HIV+ on EFV n=20  Healthy n=18 | Proguanil PK | Proguanil AUC↓43, Cmax↔ |  | Low |
| German et al. 2007 | EFV | Amodiaquine | Open label, sequential design, with washout  Amodiaquine/artesunate | Healthy n=2 | EFV PK  Amodiaquine PK | EFV PK not significantly changed  Amodiaquine AUC ↑ 115% & 302%  Desethylamodiaquine AUC↓ 8.5% & 23.7% | Significant increase in hepatic transaminases in both subjects, study terminated after 2 subjects were enrolled | Moderate |
| Huang et al. 2012 | EFV | Artemether | Open label, 2-period crossover study with washout  Artemther/lumefantrine 80/480mg BD days 1-4 and 16-28  EFV 600mg OD days 16-41 | Healthy n=12 (some PK n=10,11,7) | Artemether PK  DHA (active metabolite) PK | Small decreases in exposure (AUC↓34%, Cmax↓21%) of artemether. No statistical significance. t1/2↓44%  DHA: AUC ↓39%, Cmax ↓38%, t1/2↓13%- statistically significant. DHA:artemether ratio not significantly changed | Change in artemether AUC did not reach statistical significance. May be explained by the large artemether variability observed  in this study and the small sample size | Low |
|  |  | Lumefantrine | Open label, 2-period crossover study with washout  Artemether/lumefantrine 80/480mg BD days 1-4 and 16-28  EFV 600mg OD days 16-41 | Healthy n=11 | Lumefantrine PK  EFV PK | Lumefantrine day 7 exposure ↓46%. AUC, Cmax, t1/2↔  EFV PK↔ |  | Moderate |
| Byakika-Kibwika et al. 2012 | EFV | Artemether | Open label, one sequence crossover study with washout  Artemether/lumefantrine 80/480mg BD days 1-4 and 53-57  EFV 600mg OD + ZDV/3TC BD or TDF/FTC OD for 4 weeks. | HIV+ Malaria –  n=22 | Artemether PK  DHA PK | Artemether AUC↓79%, Cmax↓59%  DHA AUC↓75%, Cmax↓78% |  | High |
|  |  | Lumefantrine | Open label, one sequence crossover study with washout  Artemether/lumefantrine 80/480mg BD days 1-4 and 53-57  EFV 600mg OD + ZDV/3TC BD or TDF/FTC OD for 4 weeks. | HIV+ Malaria –  n=30 | Lumefantrine PK  EFV PK | Lumefantrine AUC↓56%, Cmax↓28%. Day 7 exposure↓32%  EFV ↔ |  | High |
| Hoglund et al 2014 | EFV | Artemether | Population pharmacokinetic (non-linear, mixed-effects) modelling of pooled data (Byakika-Kibwika 2012)[29, 36] | n=22 | Artemether bioavailability | Artemether bioavailability ↓71% |  | High |
|  |  | Lumefantrine | As above | n=30 | Lumefantrine CL | Lumefantrine CL↑72.6% |  | High |
| Maganda et al 2014 | EFV | Artemether  Lumefantrine | Parallel study in patients stable on ARVs, and initiating antimalarials. Clinical outcomes and PK compared between different ARV regimens or HIV+ with no ARVs at 28 days | HIV+ Malaria+  EFVn=63  NVPn=125  Control n=73 | Malaria clinical outcomes  7 day Lumefantrine plasma levels | Cumulative risk of recurrent parasitaemia >19-fold higher  in EFV-arm than control-arm  Day 7 Lumefantrine plasma conc:1,125 ng/ml (638.8-1913), 300.4 ng/ml (220.8-343.1) and 970 ng/ml (562.1-1729) for NVP-arm, EFV-arm and control-arm, respectively (P < 0.001) | No early treatment or late parasitological failure reported  No significant differences in risk of recurrent parasitaemia between patients with CD4 counts >350 compared to those with CD4 counts <350 (P = 0.204). ie between study arms | Moderate |
| Huang et al 2015 | EFV | Artemether | Parallel PK study in patients stable on ARVs, and initiating treatment for malaria. Antimalarial PK compared between different ARV regimens, and in HIV negative controls. Intensive and sparse PK sampling with population PK. | HIV+ Malaria+ paediatrics  n=91 (intensive PK), n=87 (pop PK)  (EFV n=50, LPV/r n=69, NVP n=63)  HIV - n= 51  (intensive  PK)  n=134  (pop PK) | Artemether PK  DHA PK | Artemether AUC↓56%  DHA AUC↓68% | Risk of day 28 parasitological failure: 38%  Risk of 42 day recurrent malaria: 25%  Differences in  exposure significantly  associated with  parasitologic  outcomes | High |
|  |  | Lumefantrine | As above |  | Lumefantrine PK | Lumefantrine AUC↓50%  Day 7 lumefantrine levels <175ng/ml (threshold): 85% of patients |  | High |
| Mwebaza et al. 2013 | EFV | Artemether | Parallel observational PK study in patients stable on ARVs, and initiating treatment for malaria. Antimalarial PK compared between different ARV regimens | HIV+ Malaria+ paediatrics  n=42 (EFV n=6, LPV/r n=26, NVP n=10) | Artemether PK  DHA PK | Artemether AUC↔ (compared to LPV/r or NVP containing regimens LPV/r:EFV ratio 1.5; p=0.331)  DHA AUC↔ | Antimalarial treatment failure observed in 4/6 patients taking EFV based regimen | Low |
|  |  | Lumefantrine | As above | As above | Lumefantrine PK | Lumefantrine AUC↓ (compared to NVP containing regimens, EFV:NVP 0.34, p=0.022)  Lumefantrine AUC↓6-fold (compared to LPV/r containing regimens)  Mean 7 day lumefantrine conc: (ng/ml) LPV/r 908, NVP 315, EFV 100 | As above | Low |
| Achan et al. 2012 | EFV | Lumefantrine | Parallel observational PK study in patients stable on ARVs, and initiating treatment for malaria. Antimalarial PK compared between different ARV regimens | HIV+ Malaria+ paediatrics n=65 (LPV/r), n=67 (NVP, n=25 (EFV) | 7 day lumefantrine conc. | Median lumefantrine 7 day level significantly higher in the LPV/r group than in the NNRTI group (926ng/ml [IR, 473-1910] vs. 200ng/ml [IR, 108-510], P<0.001).  Median lumefantrine 7 day level significantly higher among the patients taking NVP than patients taking EFV (388ng/ml [IR, 164-563] vs. 97ng/ml [IR, 61-124], P<0.001). | ↑serious adverse events in the LPV/r group than in the NNRTI group (5.6% vs. 2.3%, P = 0.16). Pruritus significantly more frequent in the LPV/r group, and elevated ALT levels significantly more frequent in the NNRTI group. | Low |
| Abgrall 2013 | EFV | Doxycycline | Observational PK study in patients stable on EFV containing ARVs, initiating prophylaxis with doxycycline | HIV+  EFV n=17 | EFV Ctrough | EFV Ctrough↔ |  | Low |
| Kajubi et al.  2016 | EFV | Piperaquine | PK interaction study, nested in double blind, placebo controlled studies investigating DHA-PQ for malaria prevention in pregnant women. PK of DHA-PQ was studied using an intensive design in a subset of women at 28 wks gestation with comparisons made between HIV- (no ARVs) and HIV+ (EFV-ARVs). Those randomized to DHA-PQ received a standard regimen (OD for 3 days) either monthly or bimonthly (HIV-) or monthly only (HIV+). | HIV+  n=17  HIV-  n=30  Malaria - | Piperaquine PK | Piperaquine AUC↓32-38  Piperaquine Day 7,14 and 21 levels ↓60-79% |  | Moderate |
| Soyinka et al. 2010 | EFV | Proguanil | Randomised, open label crossover, single dose PK study with washout  Proguanil 300mg alone or after 9 days EFV 400mg OD. EFV given for 11 days | Healthy n=15 | Proguanil PK  Cycloguanil PK | Proguanil AUC↑113%, Cmax↑47%, t1/2↑41%, CL↓54%  Cycloguanil AUC↓38%, Cmax↓31%, AUCcycloguanil:AUCproguanil↓68% |  | Low |
| German et al. 2009 | LPV/r | Artemether | Open label, sequential with washout  Artemether/lumefantrine 80/480 mg BD, days 1–4 and 28–31  LPV/r 400/100 mg  BD days 16–41 | Healthy  n=10 | Artemether PK  DHA (active metabolite) PK | Small decreases in exposure (AUC↓ 39%, Cmax ↓22%) of artemether. No statistical significance  DHA: AUC ↓45%, Cmax ↓36% - statistically significant  Ratio of active DHA to artemether AUC unchanged. | Change in artemether AUC did not reach statistical significance. May be explained by the large artemether variability observed  in this study and the small sample size | Moderate |
|  |  | Lumefantrine | Open label, sequential with washout  Artemether/lumefantrine 80/480 mg BD, days 1–4 and 28–31  LPV/r 400/100 mg  BD days 16–41 | Healthy  n=10 | Lumefantrine PK  LPV PK  RTV PK | Lumefantrine AUC ↑2.3 fold, Cmax ↑1.4 fold  LPV/RTV PK not significantly altered |  | High |
| Van Luin et al. 2010 | LPV/r | Atovaquone | Open label, multicentre PK study in parallel groups (HIV+ stable on ARVs containing LPV/r 400/100mg BD vs healthy volunteers)  Atovaquone/proguanil 250/100mg single dose | HIV+ on LPV/r n=19  Healthy n=18 | Atovaquone PK | AUC↓74%, Cmax↓44% |  | Moderate |
|  |  | Proguanil | Open label, multicentre PK study in parallel groups (HIV+ stable on ARVs containing LPV/r 400/100mg BD vs healthy volunteers)  Atovaquone/proguanil 250/100mg single dose | HIV+ on LPV/r n=19  Healthy n=18 | Proguanil PK | AUC↓38%, Cmax↔ |  | Low |
| Abgrall 2013 | LPV/r | Doxycycline | Observational PK study in patients stable on LPV/r containing ARVs, initiating prophylaxis with doxycycline | HIV+  LPV/r n=23 | LPV Ctrough | LPV Ctrough↔ |  | Low |
| Schippers et al  2000 | IDV | Mefloquine | PK sampling in single patient taking IDV and mefloquine concurrently, steady state  IDV 800mg TD  Mefloquine 250mg once weekly | HIV+  n=1 | IDV PK  Mefloquine PK | Co-administration did not result in sub-therapeutic or toxic levels of either drug |  | Very low |
| Kredo et al. 2016 | LPV/r | Artemether | Parallel-design safety and pharmacokinetic study. HIV-infected (malaria-negative) patients: antiretroviral-naïve and those stable on lopinavir/ritonavir-based antiretrovirals. Both groups received standard six-dose artemether-lumefantrine treatment | HIV+ (malaria-)  LPV/r n=16  ARV naive n=18 | Artemether PK  DHA PK | Artemether PK ↔  DHA AUC & Cmax ↑2-fold in the lopinavir group; this difference was no longer apparent after the last artemether-lumefantrine dose. | No safety concerns in small sample, despite large increases in lumefantrine exposures | Moderate |
|  |  | Lumefantrine |  |  | day-7 lumefantrine concentration  Adverse events related to artemether-lumefantrine. | Day -7 lumefantrine concentrations ↑~10-fold in the lopinavir group  Lumefantrine AUC ↑ 5-fold, Cmax ↑3-fold  Lumefantrine Cmax, and AUC ↑ significantly with mg/kg dose in the lopinavir group.  Similar numbers of treatment emergent adverse events and adverse reactions in both groups. No serious adverse events, no difference in QTcF- and PR-intervals |  | Moderate |
| Byakika-Kibwika et al. 2012 | LPV/r | Artemether | Parallel study in patients stabilised on LPV/r ARV therapy vs ARV naïve HIV+ patients  Artemther/lumefantrine 480/80mg single dose | HIV+  On LPV/r n=16  ARV naïve n=13 | Artemether PK  DHA PK | Artemether AUC↓43%, Cmax↓50%, CL↑67%  DHA PK↔ |  | Very Low |
|  |  | Lumefantrine | Parallel study in patients stabilised on LPV/r ARV therapy vs ARV naïve HIV+ patients  Artemther/lumefantrine 480/80mg single dose | HIV+  On LPV/r n=16  ARV naïve n=13 | Lumefantrine PK  ECG monitoring | Lumefantrine AUC↑386%, Cmax↑180%, CL↓90%  Heart rate, PR-interval, QRScomplex, QTc interval remained within normal limits in both study arms |  | Moderate |
| Hoglund et al 2014 | LPV/r | Artemether | Population pharmacokinetic (non-linear, mixed-effects) modelling of pooled data (Byakika-Kibwika 2012)[29, 36] | HIV+  On LPV/r n=16  ARV naïve n=13 | Artemether and DHA CL | Artemether CL↑32.8%  DHA CL↑143% |  | Moderate |
|  |  | Lumefantrine | As above | As above | Lumefantrine and  desbutyl‐  lumefantrine CL | Lumefantrine CL↓62%  desbutyl‐lumefantrine CL↑392% |  | Moderate |
| Huang et al 2015 | LPV/r | Artemether | Parallel PK study in patients stable on ARVs, and initiating treatment for malaria. Antimalarial PK compared between different ARV regimens, and in HIV negative controls. Intensive and sparse PK sampling with population PK. | HIV+ Malaria+ paediatrics  n=91 (intensive PK), n=87 (pop PK)  (EFV n=50, LPV/r n=69, NVP n=63)  HIV - n= 51  (intensive  PK)  n=134  (pop PK) | Artemether PK  DHA PK | Artemether AUC↓19%  DHA AUC↓16.4% | Differences in  exposure significantly  associated with  parasitologic  outcomes | High |
|  |  | Lumefantrine | As above |  | Lumefantrine PK | Lumefantrine AUC↑108%  Lumefantrine 7 day exposure↑3.4fold  10-fold reduction in lumefantrine exposure in children on EFV versus children on LPV/r |  | High |
| Mwebaza et al. 2013 | LPV/r | Artemether | Parallel observational PK study in patients stable on ARVs, and initiating treatment for malaria. Antimalarial PK compared between different ARV regimens | HIV+ Malaria+ paediatrics  n=42 (EFV n=6, LPV/r n=26, NVP n=10) | Artemether PK  DHA PK | Artemether AUC↑ (compared to NVP containing regimens, but no difference compared to EFV regimens. LPV/r:NVP ratio 2.3; p=0.028)  DHA AUC↔ | Antimalarial treatment failure observed in 2/26 patients taking LPV/r based ARV regimen | Low |
|  |  | Lumefantrine | As above | As above | Lumefantrine PK | Lumefantrine AUC↑6-fold in patients taking LPV/r regimens, compared to EFV containing regimens  Mean 7 day lumefantrine conc: (ng/ml) LPV/r 908, NVP 315, EFV 100 | As above | Low |
| Achan et al. 2012 | LPV/r | Lumefantrine | Parallel observational PK study in patients stable on ARVs, and initiating treatment for malaria. Antimalarial PK compared between different ARV regimens | HIV+ Malaria+ paediatrics n=65 (LPV/r), n=67 (NVP, n=25 (EFV) | 7 day lumefantrine conc. | Median lumefantrine 7 day level significantly higher in the LPV/r group than in the NNRTI group (926ng/ml [IR, 473-1910] vs. 200ng/ml [IR, 108-510], P<0.001). | ↑serious adverse events in the LPV/r group than in the NNRTI group (5.6% vs. 2.3%, P = 0.16). Pruritus significantly more frequent in the LPV/r group, and elevated ALT levels significantly more frequent in the NNRTI group. | Low |
| Rattanapunya et al.  2015 | LPV/r | Artesunate | Sequential crossover study in Thai adults. Period 1: standard 3-day artesunate-mefloquine (artesunate 200 mg on Days 1, 2, and 3 plus mefloquine 750 and  500 mg on Days 1 and 2, respectively); Period 2 (2 months wash-out): oral LPV/r 400 mg/100 mg BD for 14 days; Period 3: artesunate-mefloquine and LPV/r BD for 3 days | Healthy  n=15 | Artesunate PK  DHA PK | Artesunate AUC↑80%, Cmax ↑ 45%  DHA Cmax ↓46.6%, AUC0−24h↓58.2%,  AUC0−∞ ↓48.8 %  DHA:artesunate ↓72%  DHA t1/2 ↑148.5% | The 90 % CI of the GMR of Cmax for DHA, LPV, RTV, and mefloquine; AUC0−12h for RTV; AUC0−24h for artesunate and DHA; AUC0−168h for mefloquine, and  AUC0−∞ for mefloquine and RTV were outside  the acceptable bioequivalent range | Moderate |
|  |  | Mefloquine |  |  | Mefloquine PK  LPV PK  RTV PK | Mefloquine Cmax ↓19.3%, AUC0−48h ↓28.7%, AUC0−168h ↓37.1%, AUC0−∞↓35.2%  LPV AUC↔ Cmax↓22%, CL/F ↑75.4%  RTV AUC0–12h ↓44.6%, AUC0−∞↓56.3%, Cmax, of ↓54.9 %,  CL/F ↑129.1 % |  | Moderate |
| Nyunt et al 2012 | LPV/r | Quinine | 2 phase, sequential dosing PK  Study. Single dose 600mg quinine, +/- LPV/r 400mg/100mg BD for 10 days | Healthy n=12 | Quinine PK  **3-**HQ (active metabolite) PK  LPV/r PK | Quinine AUC↓50%, Cmax↓48%, Cmin↓45%  3-HQ AUC↓69%, Cmax↓69%, Cmin↓67%  LPV/r PK↔ |  | Moderate |
| Rattanapunya et al.  2015 | LPV/r | Quinine | Crossover PK study in healthy Thai adults. Period 1 (day 1): single oral dose of 600mg quinine sulfate. Period 2: LPV/r (400/100 mg) twice daily. Period 3: single quinine sulfate dose plus LPV/r twice a day. | Healthy  n=19 | Quinine PK  3-HQ PK  LPV PK  RTV PK | Quinine AUC0–48h ↓56%  AUC0–∞ ↓57%, Cmax ↓47%  3-HQ AUC0–48h ↓99%  AUC0–∞ ↓98%  Cmax ↓85%  3-HQ:Quinine ratio significantly reduced.  LPV AUC↔ Cmax ↓13%  RTV AUC ↔ Cmax ↓22% | GMR and 90% CI of AUC0–48h, AUC0–∞, and Cmax for quinine, 3-hydroxyquinine,  lopinavir, and ritonavir lay outside the bioequivalent range of 0.8–1.25. | Moderate |
| Tommasi et al. 2011 | MVC | Atovaquone | Case report of a patient with multi-drug resistant HIV infection treated with a salvage regimen of MVC 150mg BD, RAL 400mg, ETV 200mg BD and unboosted SQV 1000mg BD who started atovaquone/proguanil 250/100 mg OD prophylaxis | HIV+ n=1 | MVC PK | MVC AUC↓9% | Not likely to be clinically significant | Very Low |
|  |  | Proguanil | As above | As above | As above | As above | As above | Very Low |
| Schippers et al 2000 | NFV | Mefloquine | PK sampling in a single patient taking NFV and mefloquine concurrently, steady state  NFV 1250mg BD  Mefloquine 250mg once weekly | HIV+  n=1 | NFV PK  Mefloquine PK | Co-administration did not result in sub-therapeutic or toxic levels of either drug |  | Very low |
| Byakika-Kibwika et al. 2012 | NVP | Artemether | Open label, one sequence crossover study with washout  Artemether/lumefantrine 80/480mg BD days 1-4 and 53-57  NVP 200mg OD (2weeks), then 200mg BD + ZDV/3TC BD or TDF/FTC OD for 4 weeks | HIV+ Malaria –  n=21 | Artemether PK  DHA PK | Artemether AUC↓72%, Cmax↓61%  DHA AUC↓37%, Cmax↓45% |  | High |
|  |  | Lumefantrine | Open label, one sequence crossover study with washout  Artemether/lumefantrine 80/480mg BD days 1-4 and 53-57  NVP 200mg OD (2weeks), then 200mg BD + ZDV/3TC BD or TDF/FTC OD for 4 weeks | HIV+ Malaria –  n=28 | Lumefantrine PK  NVP PK | Lumefantrine PK ↔  NVP AUC↓46%, Cmax↓42% |  | Moderate |
| Hoglund et al 2014 | NVP | Artemether | Population pharmacokinetic (non-linear, mixed-effects) modelling of pooled data (Byakika-Kibwika 2012)[29, 36] | n=21 | Artemether bioavailability  DHA CL | Arthemether bioavailability↓66%  DHA CL↓44% |  | High |
|  |  | Lumefantrine | As above | n=28 | Lumefantrine bioavailability | Lumefantrine bioavailability ↓25% |  | Moderate |
| Huang et al 2015 | NVP | Artemether | Parallel PK study in patients stable on ARVs, and initiating treatment for malaria. Antimalarial PK compared between different ARV regimens, and in HIV negative controls. Intensive and sparse PK sampling with population PK. | HIV+ Malaria+ paediatrics  n=91 (intensive PK), n=87 (pop PK)  (EFV n=50, LPV/r n=69, NVP n=63)  HIV - n= 51  (intensive  PK)  n=134  (pop PK) | Artemether PK  DHA PK | Artemether AUC↓63.9%  DHA AUC↓29.1% |  | High |
|  |  | Lumefantrine |  |  | Lumefantrine PK | Lumefantrine AUC↔  Lumefantrine 7 day exposure↑26% |  | High |
| Maganda et al 2014 | NVP | Artemether  Lumefantrine | Parallel study in patients stable on ARVs, and initiating antimalarials. Clinical outcomes and PK compared between different ARV regimens or HIV+ with no ARVs at 28 days | HIV+ Malaria+  EFVn=63  NVPn=125  Control n=73 | Malaria clinical outcomes  7 day Lumefantrine plasma levels | Day 7 Lumefantrine plasma conc:1,125 ng/ml (638.8-1913), 300.4 ng/ml (220.8-343.1) and 970 ng/ml (562.1-1729) for NVP-arm, EFV-arm and control-arm, respectively (P < 0.001)  Cumulative risk of recurrent parasitaemia in NVP-arm not significantly higher than control | No significant differences in risk of recurrent parasitaemia between patients with CD4 counts >350 compared to those with CD4 counts <350 (P = 0.204). ie between study arms | Moderate |
| Kredo et al. 2011 | NVP | Artemether | Parallel group PK/safety study in patients stable of NVP containing ARVs, vs ARV naïve controls.  Artemether/lumefantrine 80/480mg BD for 3 days | HIV+ n=18 (on NVP) n=18 (controls) | Artemether PK  DHA PK | Artemether AUC↓74.2% Cmax↓90.3%, t1/2↔ (0-8h); AUC↔, Cmax↓56% (60-68h)  DHA PK↔ | No change in ECG or adverse event profiles | Moderate |
|  |  | Lumefantrine | Parallel group PK/safety study in patients stable of NVP containing ARVs, vs ARV naïve controls.  Artemether/lumefantrine 80/480mg BD for 3 days | HIV+ n=18 (on NVP) n=18 (controls) | Lumefantrine PK | Lumefantrine AUC↑56%, Cmax↑24%, t1/2↔  7day conc.↑82% | No change in ECG or adverse event profiles. 1/3 of ART naïve group had 7 day lumefantrine conc. below therapeutic threshold | Moderate |
| Mwebaza et al. 2013 | NVP | Artemether | Parallel observational PK study in patients stable on ARVs, and initiating treatment for malaria. Antimalarial PK compared between different ARV regimens | HIV+ Malaria+ paediatrics  n=42 (EFV n=6, LPV/r n=26, NVP n=10) | Artemether PK  DHA PK | Artemether AUC↓ (compared to LPV/r containing regimens LPV/r:NVP ratio 2.3; p=0.028)  DHA AUC↔ | Antimalarial treatment failure observed in 1/10 patients taking NVP based regimen | Low |
|  |  | Lumefantrine | As above | As above | Lumefantrine PK | Lumefantrine AUC↓ 2 fold with NVP containing regimens,  compared to LPV/r containing regimens  Lumefantrine AUC↑ with NVP compared to EFV regimens (EFV:NVP 0.34, p=0.022)  Mean 7 day lumefantrine conc: (ng/ml) LPV/r 908, NVP 315, EFV 100 | As above | Low |
| Achan et al. 2012 | NVP | Lumefantrine | Parallel observational PK study in patients stable on ARVs, and initiating treatment for malaria. Antimalarial PK compared between different ARV regimens | HIV+ Malaria+ paediatrics n=65 (LPV/r), n=67 (NVP, n=25 (EFV) | 7 day lumefantrine conc. | Median lumefantrine 7 day level significantly higher in the LPV/r group than in the NNRTI group (926ng/ml [IR, 473-1910] vs. 200ng/ml [IR, 108-510], P<0.001).  Median lumefantrine 7 day level significantly higher among the patients taking NVP than patients taking EFV (388ng/ml [IR, 164-563] vs. 97ng/ml [IR, 61-124], P<0.001). | ↑serious adverse events in the LPV/r group than in the NNRTI group (5.6% vs. 2.3%, P = 0.16). Pruritus significantly more frequent in the LPV/r group, and elevated ALT levels significantly more frequent in the NNRTI group. | Low |
| Parikh et al  2015 | NVP | Arthemether | Parallel PK study with historical controls. Patients who were taking NVP-based ARVs received ZDV-3TC-NVP BD. Following PK sampling, participants received cartemether-lumefantrine 80/480 mg BD for 3 days, alongside ARVs. The control group received artemether-lumefantrine alone | HIV+ NVP  Malaria -  n=11  Healthy n=16 | Artemether PK  DHA PK  NVP PK | Artemether AUC↓68%  DHA↔  NVP PK ↔ |  | Low |
|  |  | Lumefantrine |  |  | Lumefantrine PK | Lumefantrine AUC↓49%  25% of participants who received NVP-based ART had day 7 concentrations below therapeutic thresholds (175 or 280 ng/ml) |  | Low |
| Chijioke-Nwauche et al. 2013 | NVP | Lumefantrine | Observational PK; unmatched case-control design, in patients initiating antimalarial treatment with artemether/lumefantrine 4 tablets BD for 3 days, with or without NVP use | Patients with malaria  HIV+ (NVP) n=68  HIV - n=99 | Day 7 lumefantrine blood levels  Parasite carriage day 3 and day 28  posttreatment | Median 7 day lumefantrine conc. in HIV-negative group: 2.75 µM  vs HIV-positive (NVP)  Group: 3.55µM (*P*=0.0011) | Weak association  between day 3 parasitemia and higher lumefantrine  concentrations (*P* = 0.021), suggesting that  ↑ lumefantrine bioavailability in NVP recipients was not improving outcomes. | Very Low |
| Scarsi et al 2014 | NVP | Amodiaquine | Parallel group PK study, in patients stable on NVP containing ARVs, vs ARV naïve controls.  Artesunate-amodiaquine 200/600mg OD for 3 days | HIV +  n=10 (on NVP)  n=11 (controls) | Amodiaquine PK  DEAQ PK | Amodiaquine AUC↓28.9%, Cmax↔, CL ↑50%, Vd↑2.5fold  DEAQ AUC↓32.7%, Cmax↔ | Considerable inter-patient variability for amodiaquine PK parameters | Low |
| Fehintola et al 2012 | NVP | Artesunate | Parallel group PK study, in patients stable of NVP containing ARVs, vs ARV naïve controls.  Artesunate-amodiaquine 200/600mg OD for 3 days | HIV +  n=10 (on NVP)  n=11 (controls) | Artesunate PK  DHA PK | Artesunate AUC↑45%, CL↓50%, trend↓t1/2. Other PK↔  DHA t1/2↓, other PK↔ DHA:artesunate AUC↓ | No treatment-limiting adverse events. Authors conclude that overall exposure to artesunate/DHA similar in both groups | Low |
| Abgrall et al 2013 | NVP | Doxycycline | Observational PK study in patients stable on NVP containing ARVs, initiating prophylaxis with doxycycline | HIV+  NVP n=10 | NVP Ctrough | NVP Ctrough↔ |  | Low |
| Soyinka et al. 2009 | NVP | Quinine | Open label, randomised, crossover design, with washout.  Single dose quinine 600mg ± steady-state NVP 200mg BD for 12 days | Healthy n=14 | Quinine PK  3-HQ PK | Quinine AUC↓33%, Cmax ↓ 36%  3-HQ AUC↑30%; Cmax↑25% |  | Low |
| Uriel et al. 2011 | NVP | Quinine | Case report. A patient was acutely unwell and started on quinine, co-amoxiclav and clarithromycin. Current ARVs were confirmed as ABC/3TC and NVP | n=1 | Clinical efficacy of quinine | *P.falciparum* parasitaemia increased from 1% to 2.5% in the presence of quinine taken with NVP. Induction of quinine CYP3A4 metabolism by NVP possible. |  | Very Low |
| Kayentao et al 2014 | NVP | Quinine | Observational PK study in pregnant women taking NVP based ARVs and requiring antimalarial treatment. Quinine sulphate 600mg TDS day 0-6 | Pregnant HIV+  Malaria+  n=6 with NVP in plasma n=1 with no NVP in plasma) | Quinine PK  Malaria cure | 4-fold↑ ratio of 3-HQ:quinine in the presence of NVP  All patients achieved complete cure. | Study underpowered to assess impact of NVP on quinine treatment efficacy. | Very low |
| Tomassi et al. 2011 | RAL | Atovaquone | Case report of a patient with multi-drug resistant HIV infection treated with a salvage regimen of MVC 150mg BD, RAL 400mg, ETV 200mg BD and unboosted SQV 1000mg BD who started atovaquone/proguanil 250/100 mg OD prophylaxis | n=1 | RAL PK | RAL AUC↓23% |  | Very Low |
|  |  | Proguanil | As above | As above | As above | As above | As above | Very Low |
| Morris et al. 2012 | RTV | Artesunate | Randomised parallel group study  **A** RTV 100mg BD day 1-17  Pyronidine/artesunate (weight based 540/180mg or 720/240mg) once daily day 8-10  **B** Pyronidine/artesunate as above, day 1-3 | Healthy  **A** n=17  **B** n=16 | Artesunate PK  DHA PK  RTV PK | Artesunate AUC↑27%, Cmax↔  DHA AUC↓38%, Cmax↓27%  RTV exposure↑3.2fold | ↑Liver enzymes (4 subjects arm A discontinued) | Moderate/High |
|  |  | Pyronaridine | As above | Healthy  **A** n=17  **B** n=16 | Pyronaridine PK  RTV PK | Pyronaridine PK↔  RTV exposure↑3.2fold | ↑Liver enzymes (4 subjects arm A discontinued) | Moderate/High |
| Khaliq et al 2001 | RTV | Mefloquine | Part 1. Open label sequential design with washout, steady state RTV.  RTV 200mg BD; mefloquine 250mg OD 3/7, then weekly 4/52  Part 2. Open label, steady state mefloquine with RTV single dose  RTV 200mg; mefloquine 250mg OD 3/7, then weekly 2/52 | Healthy  Part.1 n=11  Part.2 n=12 | RTV PK  Mefloquine PK | Part 1. No significant changes to mefloquine AUC and Cmax (<7% changes)  RTV AUC ↓31%, Cmax↓36%  Part 2. No significant changes to single dose RTV PK (<8% changes to AUC, Cmax) | RTV 200mg BD dose used, difficult to assess in context of boosting  Mefloquine loading not used in clinical practice for malaria treatment or prophylaxis | Low |
| Soyinka et al. 2010 | RTV | Quinine | 3 period sequential PK study with washout.  Quinine 600mg single dose, followed by RTV 200mg BD for 9 days, quinine 600mg single dose with 15^th^ dose of RTV | Healthy n=10 | Quinine PK 3-HQ PK  RTV PK | Quinine AUC↑341%, Cmax↑284%, CL↓77%, t1/2↑20%  3-HQ AUC↓60%, Cmax↓50%  AUC3-HQ:AUCQuinine↓90%  RTV AUC↑21%, Cmax↑15%, Cmin↑66%, t1/2↑32% |  | Low/Very Low |
| Tommasi et al. 2011 | SQV | Atovaquone | Case report of a patient with multi-drug resistant HIV infection treated with a salvage regimen of MVC 150mg BD, RAL 400mg, ETV 200mg BD and unboosted SQV 1000mg BD who started atovaquone/proguanil 250/100 mg OD prophylaxis | n=1 | SQV PK | SQV AUC↑274% | Single case report. No adverse events or laboratory abnormalities | Very Low |
|  |  | Proguanil | As above | As above | As above | As above | As above | Very Low |
| Lee et al  1996 | ZDV | Atovaquone | Open-label, randomised, sequential design.  ZDV 200mg 8 hourly  Atovaquone 750mg 12 hourly | HIV +  n=14 | ZDV PK  ZDV-glucuronide PK  Atovaquone PK | ZDV AUC ↑33%, CL ↓34%.  ZDV-glucuronide AUC ↓  Significant ↓ AUC ZDV-glucuronide : AUC ZDV  ZDV-glucuronide Cmax ↓ significantly  ZDV Cmax unchanged  Atovaquone PK unchanged | ATQ dose studied is for PCP; higher than that used for malaria treatment or prophylaxis | Low |
| Jacobson et al. 1996 | ZDV | Pyrimethamine | Open-label study, with addition of pyrimethamine to steady state ZDV, in patients receiving ≥500mg daily.  ZDV 100mg before and after pyrimethamine.  Pyrimethamine 200mg loading dose, then 50mg OD 3/52 | HIV +  n=10 | ZDV PK | No significant difference in ZDV AUC or Cmax | Pyrimethamine dose and duration relates to cerebral toxoplasmosis; limited applicability to malaria | Moderate |
